# Experimental data on water soluble polymers thermal and hydrolytic stability, reactivity ratios of monomers and Frr calculation for thermally stable preformed particle gels therefrom

**DOI:** 10.1016/j.dib.2021.107357

**Published:** 2021-09-08

**Authors:** Buddhabhushan Salunkhe, Thomas Schuman, Ali Al Brahim, Baojun Bai

**Affiliations:** a Department of Chemistry, Missouri University of Science and Technology, 335 Schrenk Hall, Rolla, MO 65409, USA; b Petroleum Engineering, Missouri University of Science and Technology, Rolla, MO 65409, USA

**Keywords:** Thermal stability of polymers, Hydrolytic stability of polymers, Viscosity measurements, Thermogravimetric analysis (TGA), Reactivity ratio, Residual resistance factor (Frr)

## Abstract

Experimental data on water soluble polymer thermal and hydrolytic stability in acidic, neutral and basic pH conditions in aqueous solution is presented. Thermal and hydrolytic stability of polymer aqueous solutions were monitored in variable pH medium by aging at 130 °C temperature for different aging time. Polymer viscosity measurements were performed periodically for 3 months. Furthermore, this data can serve as a basis of monomer selection for thermally stable hydrogels for applications like oil recovery, hydrogel coatings for steam sterilized medical devices and other applications. Reactivity ratios were determined for monomers associated to the most stable polymers using ^1^H NMR analysis. Monomer reactivity ratios for DMA (M_1_) and NaSS (M_2_) depicted to be *r*_1_ = 0.031 and *r*_2_ = 5.379 using Fineman-Ross method and *r*_1_ = 0.028 and *r*_2_ = 5.495 using Kelen-Tudos method. The performance of preformed particle gels developed based on these monomers, in plugging the open fractures is explained using residual resistance factor (Frr) calculation.

## Specifications Table

**Table:** 

Subject	Chemistry
Specific subject area	Polymer thermal and hydrolytic stability in aqueous solutions, reactivity ratios of monomers, residual resistance factor (Frr) calculation
Type of data	Table Image Graph Figure
How data were acquired	Polymer thermal and hydrolytic stability was determined by viscosity measurements on Brookfield DV3T viscometer. For reactivity ratio, copolymer composition was analysed using ^1^H NMR technique. Residual resistance factor (Frr) calculations were explained based on lab scale core flooding test performed in sandstone core.
Data format	Raw Analyzed
Parameters for data collection	Change in viscosity as a function of time for polymer thermal and hydrolytic stability. For reactivity ratios, copolymer composition as a function of comonomers feed ratio was studied using Fineman-Ross method and Kelen-Tudos method. Frr, calculations were done based on pressure gradient values obtained during a core-flooding test.
Description of data collection	For hydrolytic thermal stability of polymer aqueous solutions, viscosity at 23 °C was determined for samples that were exposed to 130 °C temperature for variable time in acidic, basic and neutral pH. The change in viscosity was correlated to polymer molecular weight change and determine polymer backbone stability. For reactivity ratios, polymerization was conducted at different co-monomers feed, and by collecting the copolymer at early stage of polymerization (preferably less than 10% conversion) and analysed using ^1^H NMR. For Frr calculation, permeability values were obtained from stable pressure gradient values following Darcy law.
Data source location	Institution: Missouri University of Science and Technology City/Town/Region: Rolla, Missouri Country: United States of America
Data accessibility	With the article
Related research article	B. Salunkhe, T. Schuman, A A Brahim, B. Bai, Ultra-High Temperature Resistant Preformed Particle Gels for Enhanced Oil Recovery, Chem. Eng. J., Volume 426, 15 December 2021, 130712 [https://doi.org/10.1016/j.cej.2021.130712].

## Value of the Data

• The dataset represents viscosity measurements data to determine thermal and hydrolytic stability when polymer aqueous solutions were prepared in acidic, neutral and basic medium.
• The data on reactivity ratios of p-Sodium styrene sulfonate and N, N’-Dimethylacrylamide was reported and can be beneficial for researchers who are interested in synthesizing copolymer compositions based on these monomers for variety of applications. This is the first report of reactivity ratio data for this monomer combination.
• Residual resistance factor (Frr) calculation are critical and small errors can give rise to large errors in Frr values. Dataset for Frr explained herein can aid a direction for petroleum engineers to conduct experiments to understand plugging efficiency of open fractures.

## 1 Data Description

**Fig. 1:**
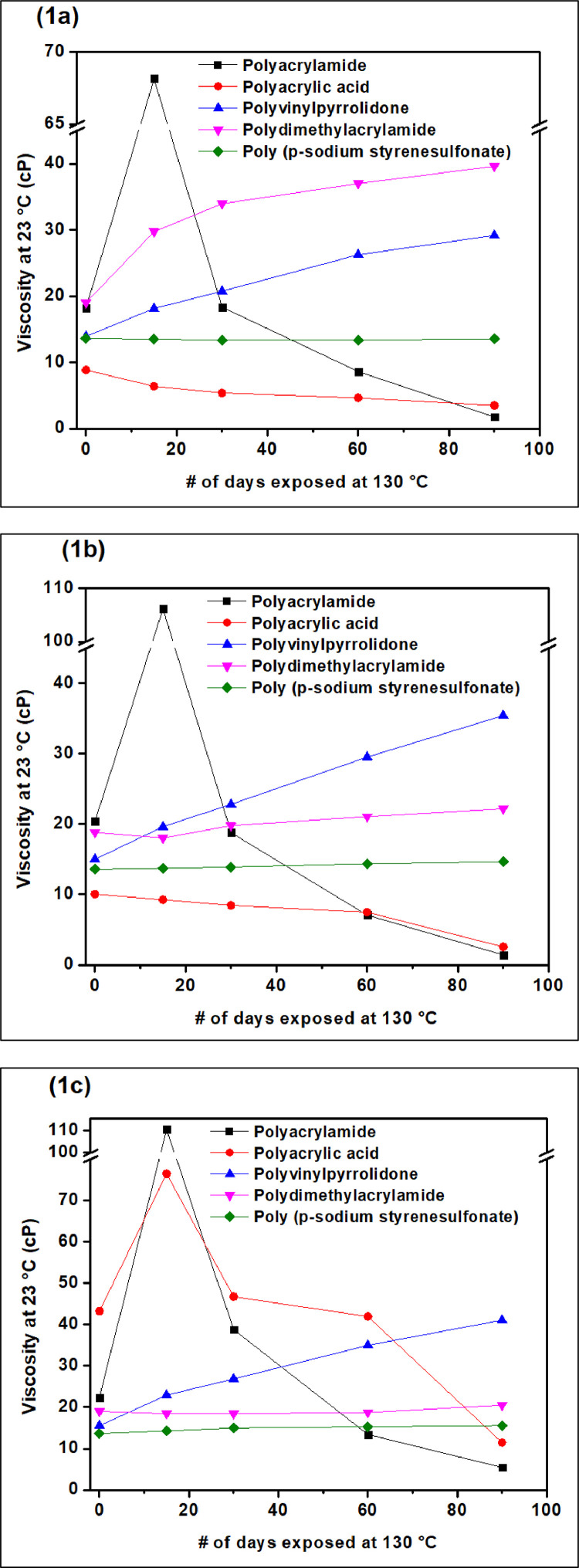
Thermal and hydrolytic stability of polymer aqueous solutions when exposed at 130 °C for different timeframes. The plot represents viscosity (cP) for polymer aqueous solutions prepared in acidic solution of pH=2 (fig. 1a), neutral pH=7 (Fig. 1b) and basic pH=13 (Fig. 1c) as a function of aging time (days).

Data for thermal and hydrolytic stability of a polymer aqueous solutions is represented in Fig. 1. The dataset represents the viscosity values of polymer aqueous solutions recorded as a function of time (# of days exposed at 130 °C) when prepared in medium of pH 2 (acidic), pH 7 (neutral) and pH 13 (basic). The obtained viscosity values for polyacrylamide and polyacrylic acid depicts that these polymers are hydrolytically and thermally unstable at 130 °C in all pH media. For Polyvinylpyrrolidone polymer, viscosity increase as a function of time in all pH media was noted, which can be attributed to partial hydrolysis of polymers. Polydimethylacrylamide showed increase in viscosity as a function of time under acidic condition, on the other hand, under neutral and basic condition, less significant changes to the initial reported viscosity values were observed. Poly (sodium styrenesulfonate) polymer solutions showed almost similar viscosity values over the period of exposure under all pH conditions, depicting the thermal and hydrolytic stability of this polymer. Actual values for all polymer solutions are reported in attached excel file. This dataset can be useful in screening of polymers for applications involving variable moisture, pH and temperature conditions.

**Fig. 2:**
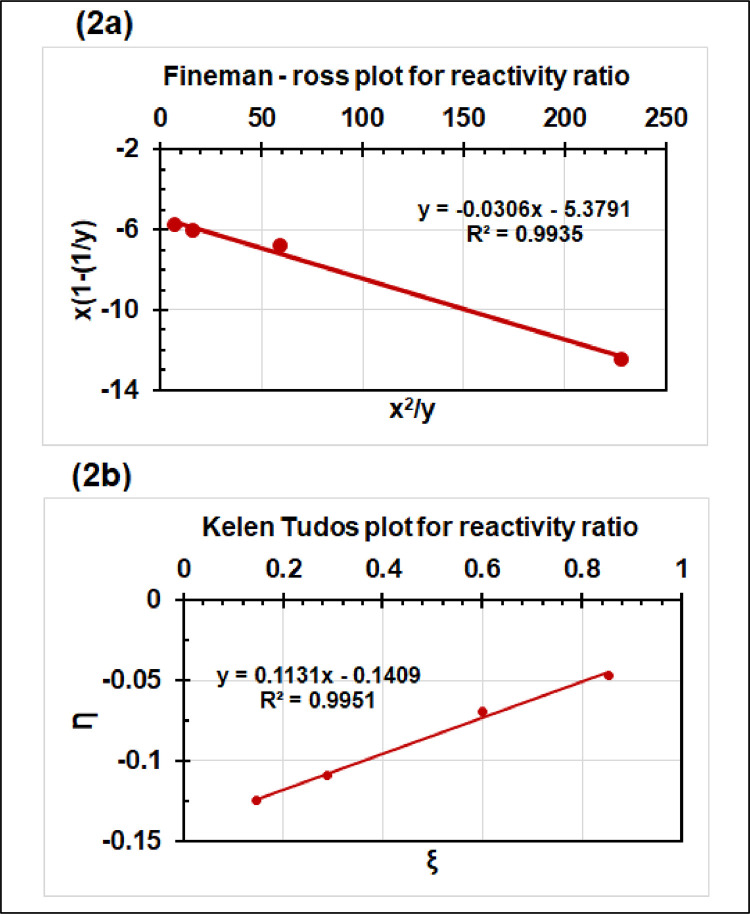
Reactivity ratios for monomers dimethylacrylamide [monomer 1] and p-(sodium styrenesulfonate) [monomer 2] using Fineman-Ross method (Fig. 2a) and Kelen-Tudos method (Fig. 2b).

Fig. 2 shows the reactivity ratio plots for monomers dimethylacrylamide and p-(sodium styrenesulfonate) using Fineman-ross method (Fig. 2a) and Kelen-Tudos method (Fig. 2b). The monomer reactivity ratios estimated to be *r*_1_ = 0.031 and *r*_2_ = 5.379 using Fineman-Ross method and *r*_1_ = 0.028 and *r*_2_ = 5.495 using Kelen-Tudos method where M_1_ and M_2_ monomers are for DMA and NaSS, respectively. These parameters indicate that NaSS has very high reactivity than that of DMA.

**Fig. 3:**
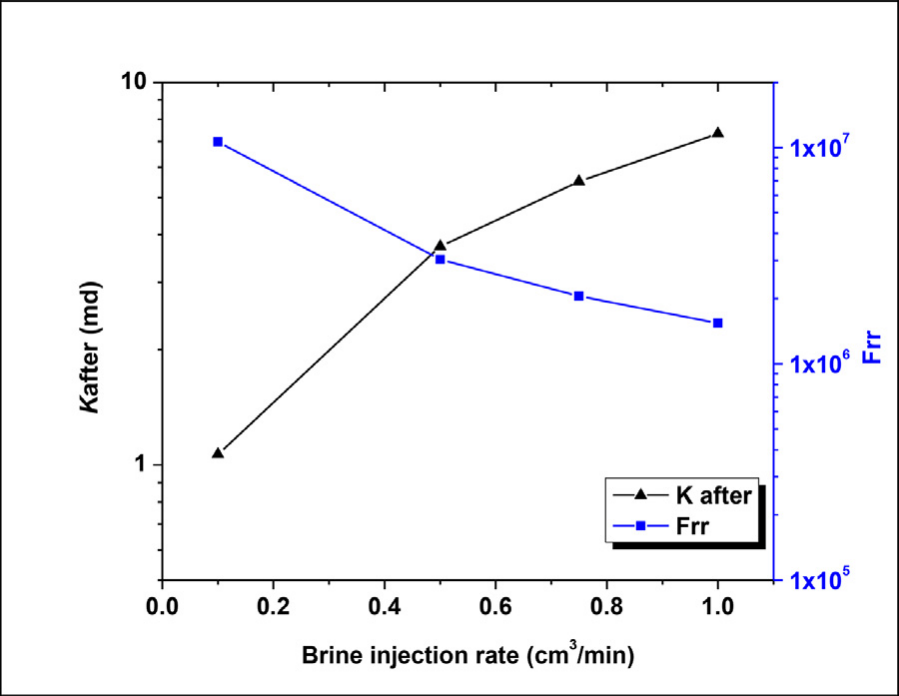
Residual resistance factor (Frr) and fracture permeability (*K*after) at variable brine injection rates.

The Residual resistance factor (F_rr_) is an important parameter to confirm plugging efficiency performance data obtained in core-flooding test. F_rr_ was calculated in order to determine the extent of the permeability reduction to water of the fractured core after the gel placement. Residual resistance factor (Frr) and fracture permeability (*K*after) at variable brine injection rates is shown in Fig. 3. The Frr values decreased with the increase in brine injection flow rates, likely indicating that the chase water created a path through or around the packed gel since no gel washout was observed with the increase in brine injection rates (explanation can be found in our CEJ paper).

## 2 Materials, Methods, Sample Preparation

### 2.1 Materials

Polyacrylamide, Polyacrylic acid, Polyvinylpyrrolidone, Polydimethylacrylamide, Poly (sodium styrenesulfonate) commercial samples through Sigma-Aldrich, deuterium dioxide, Sodium styrenesulfonate (NaSS), N, N’-dimethylacrylamide (DMA), VA-044 initiator.

### 2.2 Sample preparation for thermal and hydrolytic stability

Polymer aqueous solutions were prepared in different pH medium as acidic, neutral and basic in deionized water. Acidic solution of pH=2 was prepared using 0.1 M HCl and basic solution of pH=12 was prepared using 0.1 M NaOH along with neutral pH=7 solution. Polymer aqueous solutions prepared in these solutions, at a concentration, just enough to get measurable value on Brookfield viscometer. Glass pressure tubes were used for testing, where sample was transferred through canula transfer and sealed under argon atmosphere. These glass tubes were then kept in oven maintained at 130 °C and periodic measurements viscosity measurements were performed.

### 2.3 Monomer reactivity ratio determination

The polymerization of NaSS and DMA were conducted at different monomer feed ratio using VA-044 initiator in D_2_O. The polymerization reaction was conducted under argon inert atmosphere at 40 °C. After specific time, ^1^H NMR spectrum was collected on 400 MHz NMR system, where monomer conversions were determined by monitoring the change in the intensity of peaks associated with vinylic protons of the monomers.

### 2.4 Residual resistance factor (Frr) calculation for coreflooding experiments

The Frr is defined as the ratio of the mobility of water before the gel placement to the mobility of water after the gel placement [1].

$$F_{\mathrm{rr}}\;=\;\frac{\lambda_{b}}{\lambda_{a}}=\frac{K_{b}/\mu_{w}}{K_{a}/\mu_{w}}={\left(\frac{\Delta P_{a}}{\Delta P_{b}}\right)}_{q}$$

Where $K_{b}$ represents the initial permeability of the fractured core including both the matrix and the fracture parts, and Ka is the permeability of the fractured core to brine after the HT-PPG placement. $\mu_{w}$ is the viscosity of the injected brine.

By applying Darcy's law

$$q=\frac{KA\Delta P}{\mu L}$$

Where q is the volumetric injection flow rate (cc/sec), A is the cross sectional area of the fractured core (cm^2^), ΔP is the pressure drop along the fractured core (atm), µ is the viscosity of the brine and L is the length of the fractured core. The Frr can be expressed in terms of pressure gradient along the fractured core once the steady state condition is achieved as a function of the injection flow rate. The data file for pressure gradient values obtained during a core-flood experiment is attached. These pressure gradient values were used for further calculations.

The initial permeability of the fractured core $K_{b}$ = 11313 Darcy.

The pressure drop across the fractured core during the first water flooding was calculated using the following equation of the flow through the parallel plate model

$$\Delta P_{b}=\frac{12\mu Lq\;}{9.86\;X\;10^{7}\;{w_{f}}^{3}\;w_{h}}$$

Where, $w_{f}$ is the fracture width and $w_{h}$ is the fracture height for core used in core-flooding experiment.

## Ethics Statement

The authors declare that they have followed the general ethics rules of scientific research performance and publishing.

## CRediT authorship contribution statement

**Buddhabhushan Salunkhe:** Methodology, Investigation, Data curation, Visualization, Validation, Writing – original draft. **Thomas Schuman:** Supervision, Writing – review & editing. **Ali Al Brahim:** Methodology, Investigation, Data curation, Visualization. **Baojun Bai:** Supervision.

## Declaration of Competing Interest

The authors declare that they have no known competing financial interests or personal relationships which have or could be perceived to have influenced the work reported in this article.
